# An Efficient Intestinal Organoid System of Direct Sorting to Evaluate Stem Cell Competition in Vitro

**DOI:** 10.1038/s41598-019-55824-1

**Published:** 2019-12-30

**Authors:** Yuki Fujimichi, Kensuke Otsuka, Masanori Tomita, Toshiyasu Iwasaki

**Affiliations:** Radiation Safety Research Center, Nuclear Technology Research Laboratory, Central Research Institute of Electric Power Industry (CRIEPI), 2–11–1 Iwado Kita, Komae, Tokyo 201–8511 Japan

**Keywords:** Tissue engineering, Intestinal stem cells

## Abstract

Stem cell competition could shed light on the tissue-based quality control mechanism that prevents carcinogenesis. To quantitatively evaluate stem cell competition *in vitro*, we developed a two-color intestinal organoid forming system. First, we improved a protocol of culturing organoids from intestinal leucine-rich-repeat containing G-protein-coupled receptor 5 (Lgr5)- enhanced green fluorescent protein (EGFP)^high^ stem cells directly sorted on Matrigel without embedding. The organoid-forming potential (OFP) was 25% of Lgr5-EGFP^high^ cells sorted at one cell per well. Using this culture protocol with lineage tracing, we established a two-color organoid culture system by mixing stem cells expressing different fluorescent colors. To analyze stem cell competition, two-color organoids were formed by mixing X-ray-irradiated and non-irradiated intestinal stem cells. In the two-color organoids, irradiated stem cells exhibited a growth disadvantage, although the OFP of irradiated cells alone did not decrease significantly from that of non-irradiated cells. These results suggest that stem cell competition can be evaluated quantitively *in vitro* using our new system.

## Introduction

Homeostasis of the small intestine epithelium is sustained by self-renewing stem cells that are located at the bottom of crypts. The small cycling cells between Paneth cells are known as crypt base columnar (CBC) cells^1^. Leucine-rich-repeat containing G-protein-coupled receptor 5 (Lgr5) is one of the molecular markers for CBC cells^2^. Genetic lineage tracing experiments revealed that Lgr5^+^ CBC cells were multipotent stem cells because they were self-renewing and gave rise to all types of intestinal epithelial cells^3^. The expression of Lgr5 was observed strongly in CBC cells and decreased gradually along with their differentiation. The differentiated cells migrated from crypts to the villus and became functional cells.

Lgr5^+^ stem cells divide symmetrically, and daughter cells are stochastically destined to retain stemness in niches, or not, by neutral competition to maintain homeostasis of the stem cell pool^4^. However, neutral competition can be disturbed by the emergence of mutated Lgr5^+^ stem cells and, consequently, cause tumorigenesis after the stem cell pools are predominantly filled by mutated stem cells^5^. In contrast, cell competition is a well-known phenomenon that can eliminate pre-cancerous cells, in some cases, when they are surrounded by normal cells^6^. Cell competition between normal and oncogenic transformed epithelial cells was recently reported^7,8^, whereas non-neutral competition between two isogenic stem cells has not yet been reported, despite being an important issue in both development and disease^9^.

The study of intestinal stem cells has been accelerated using three-dimensional *in vitro* enteroids embedded in Matrigel^10^. Intestinal organoids not only contain stem cells, but also all types of intestinal epithelial cells. Previously, we employed an organoid-forming assay to estimate the survival rate of stem cells exposed to ionizing radiation^11^. This assay enabled us to assess the sensitivity of small intestinal stem cells to low-dose radiation. However, the organoid-forming efficiency was insufficient (1.7–3.3%) to assess stem cell competition quantitatively; this might result from damage caused by enzymatic dissociation or culture stress. In the current study, we optimized a protocol of culturing organoids from intestinal stem cells highly expressing Lgr5-enhanced green fluorescent protein (Lgr5-EGFP^high^) that were directly sorted on medium containing Matrigel without embedding in Matrigel. Based on this protocol, we established a high-efficiency system that enabled the quantitative evaluation of stem cell competition between irradiated and non-irradiated Lgr5^+^ stem cells.

## Results

### Improvement of organoid-forming efficiency (OFE) by optimizing the culturing medium contents

**Figure 1:**
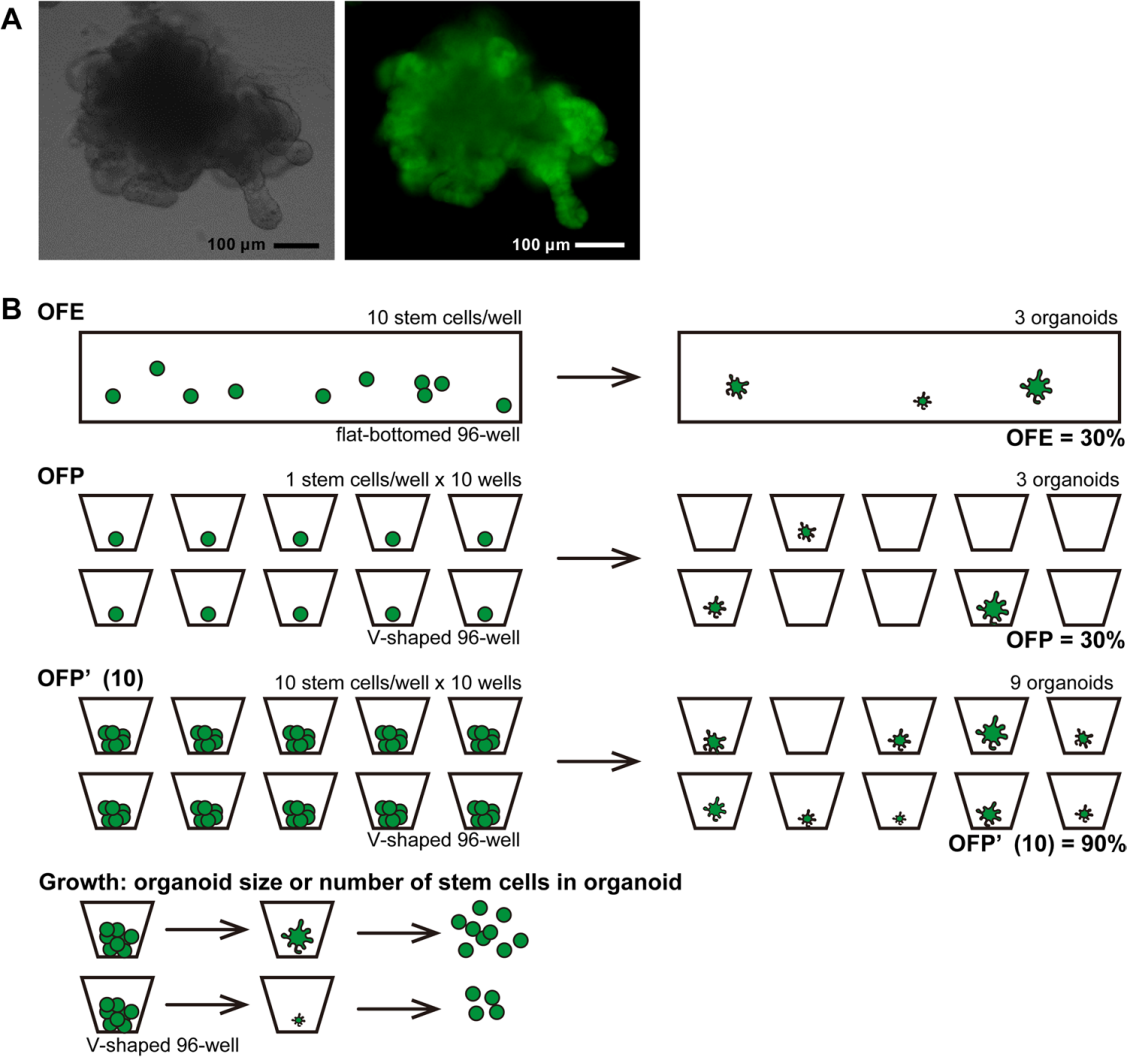
A duodenum organoid and conceptual diagram of the definitions used to evaluate organoid-forming potential. (**A**) Representative images of an organoid. Left is a bright field image and right is a fluorescent image of Lgr5-enhanced green fluorescent protein in an organoid at Day 14.

We established a high-efficiency organoid culture protocol that could generate an organoid from a single Lgr5-EGFP^high^ stem cell by direct sorting into medium containing Matrigel in flat-bottomed plates (Fig. 1A). In this method, it was not necessary to concentrate intestinal stem cells by centrifugation after sorting, then mixing and embedding them in Matrigel. To evaluate the organoid forming capacity of stem cells, the OFE was calculated as a percentage of the number of organoids per number of plated stem cells (Fig. 1B).

**Figure 2:**
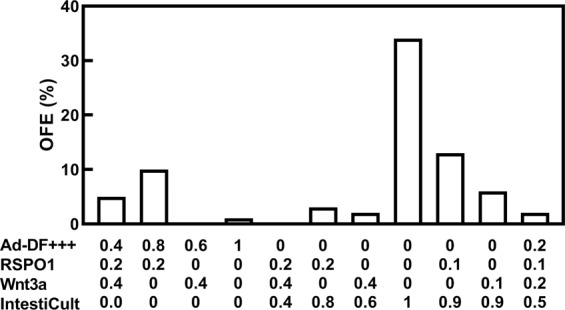
Organoid-forming efficiency (OFE) of various culture media. OFEs for each basal medium (n = 1). Ad-DF^+++^ is advanced Dulbecco’s modified Eagle’s/F12 medium supplemented with GultaMax, 1 M HEPES, and penicillin/streptomycin. RSPO1 is R-spondin1.

The OFE differed greatly depending on the type of medium and supplements provided (Fig. 2). The highest OFE was obtained using IntestiCult without conditioned media containing Wnt3a and/or R-spondin1, which are factors secreted by Paneth cells, although Noggin and epidermal growth factor were added to IntestiCult as described in Methods. The organoids showed fine shapes with budding. In 20% Wnt3a conditioned medium, the concentration of Wnt3a was 15 ng/mL as measured by an enzyme-linked immunosorbent Assay (ELISA) (Table S1). We were unable to detect R-spondin1 by ELISA in the R-spondin1 conditioned medium used in this study. In addition, neither Wnt3a nor R-spondin1 could be detected by ELISA in IntestiCult. The medium with Matrigel was maintained at 4 °C or on ice during cell sorting. Stem cells in the medium were then incubated at 37 °C to form organoids. The OFE of Lgr5-EGFP^high^ single cells by direct sorting reached 34% at Day 6 (Fig. 2). In addition, Lgr5-EGFP^high^ cells isolated enzymatically from organoids could form second and third organoids at high efficiency (>60%) (data not shown).

### Definition of organoid-forming potential (OFP) for evaluating the potential of single stem cells to form organoids

**Figure 3:**
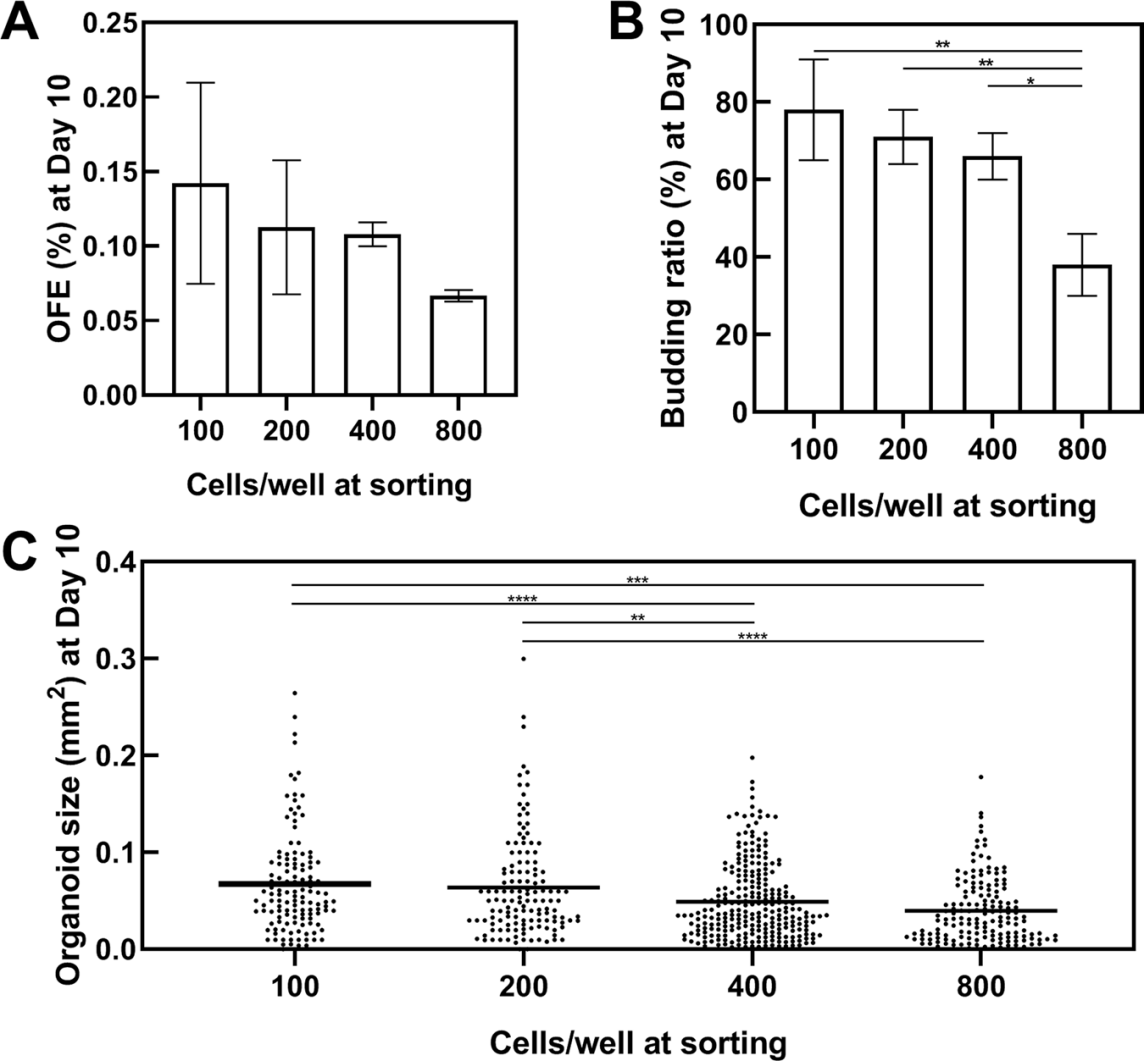
Organoid formation is affected by the number of cells. (**A**) Organoid-forming efficiency (OFE) depends on the seeded cell concentration (n = 3). There is no statistically significant difference (*P* > 0.05, Tukey-Kramer). (**B**) The budding ratio depends on the seeded concentration (n = 3) (*P < 0.05, **P < 0.01, Tukey-Kramer). (**C**) Organoid size evaluated by area (mm^2^). The average areas of organoids are shown as black bars (**P < 0.01, ***P < 0.001, ****P < 0.0001, Tukey-Kramer).

**Figure 4:**
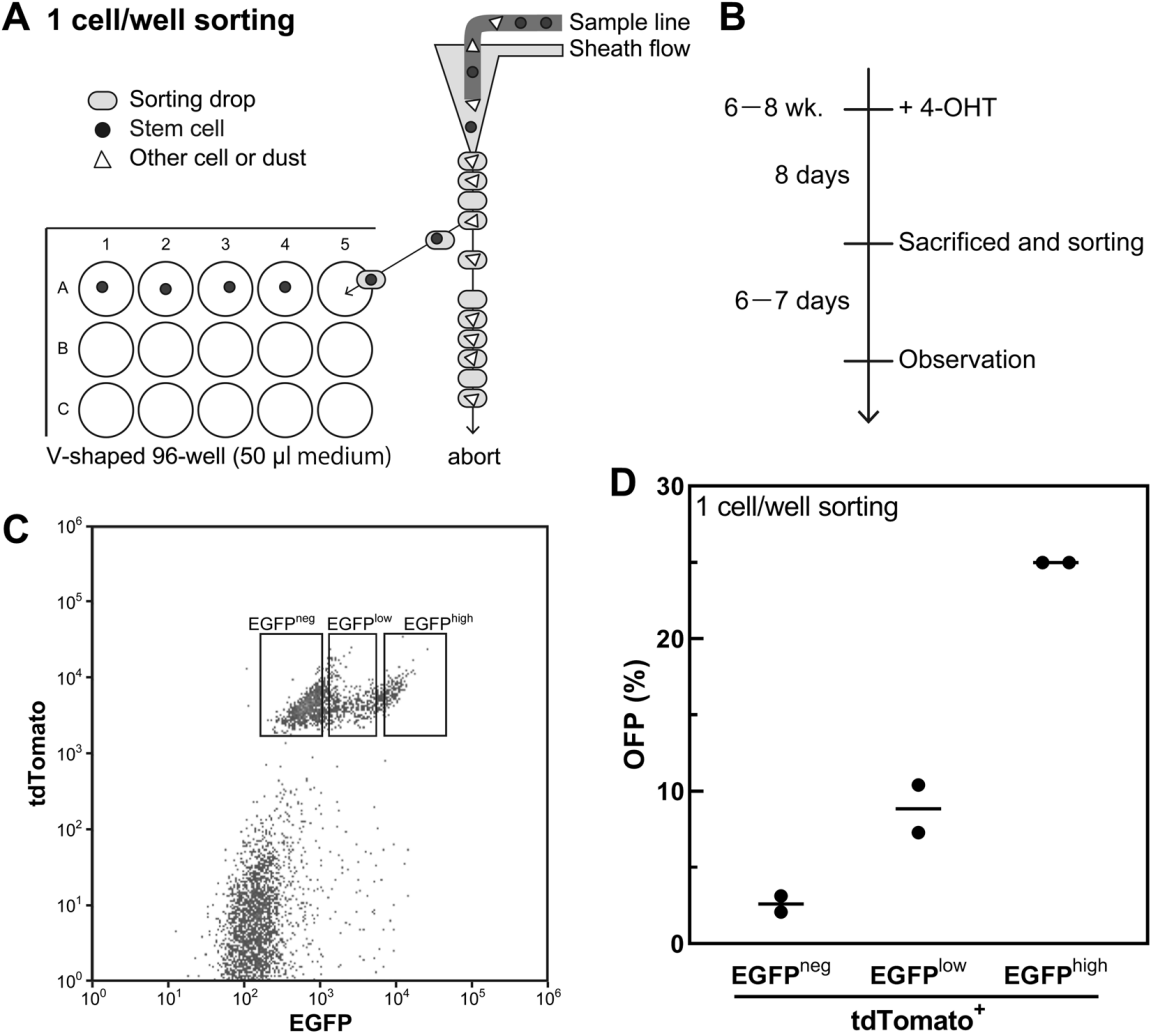
One cell per well direct sorting. (**A**) Schematic of the method used for one cell per well direct sorting. (**B**) Organoid-forming procedure. (**C**) Sorting gate for (**D**). (**D**) Organoid-forming potential (OFP). Average OFPs are shown as black bars. EGFP, enhanced green fluorescent protein; 4-OHT, 4-hydroxytamoxifen.

The OFE decreased with an increasing number of plated stem cells per well (Fig. 3A) because many organoid-initiating stem cells contacted each other and created only a single organoid under high density conditions. Additionally, too many cells in a well inhibited cell proliferation; therefore, organoid areas and budding rates also decreased with an increasing concentration of cells (Fig. 3B,C). These results suggest that this method cannot assess the OFE and growth rate accurately when numerous stem cells are sorted into 10% Matrigel-containing medium with flat-bottomed 96-well plates because they are greatly affected by the concentration of cells. Thus, we constructed a “one cell/well direct sorting” method with 1% Matrigel using plates with V-shaped wells. For the one cell/well direct sorting method, organoid medium (50 μL) was added into each well of a 96-well plate (Fig. 4A,B). Then, Lgr5-EGFP^high^ cells were plated into the wells (one cell/well). To evaluate the organoid forming capacity of stem cells, the OFE (%) was calculated as the number of organoids per number of plated stem cells when a large number of stem cells were sorted into a well. We newly defined the OFP (%) as a percentage of the number of organoids per number of plated stem cells when one cell was sorted into a well to avoid confusion between the two organoid formation methods (Fig. 1B). The OFPs of Lgr5-EGFP^high^, Lgr5-EGFP^low^, and Lgr5-EGFP^neg^ were 25, 9, and 3%, respectively (Fig. 4C,D).

### Quantitative analysis for mixed organoids derived from two different stem-cell populations

**Figure 5:**
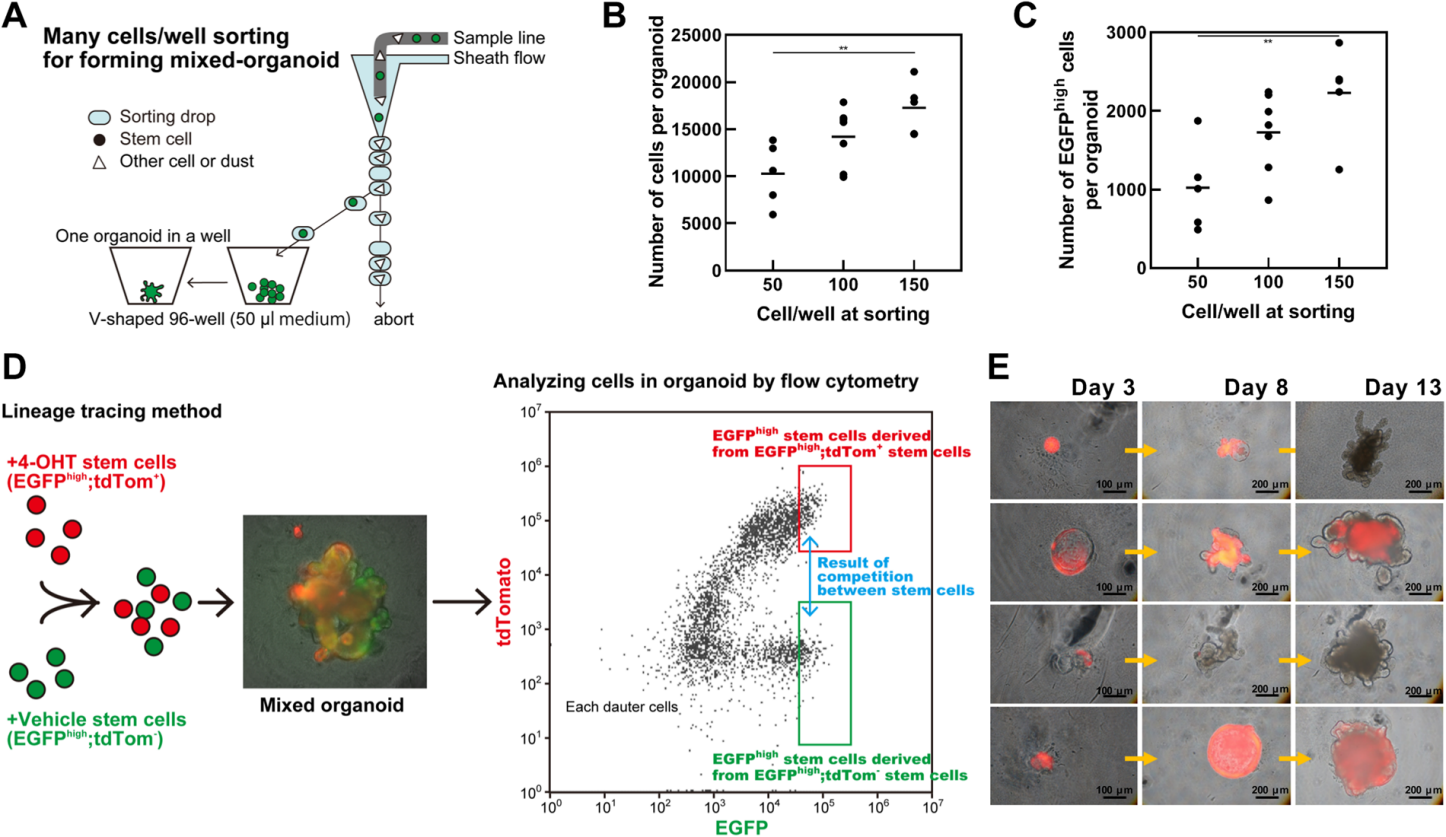
Strategy of forming mixed organoids. (**A**) Schematic of the “mixed culture by direct sorting” procedure for forming one organoid derived from many stem cells. (**B**,**C**) Numbers of total (**B**) and Lgr5-enhanced green fluorescent protein (EGFP)^high^ (**C**) cells in organoids at Day 9 depend on the initial number of stem cells. Closed circles indicate individual organoids and black bars show their average (**P < 0.01, Tukey-Kramer). (**D**) Strategy of forming mixed organoids derived from two-color stem cells and their analysis. (**E**) Representative images of organoid growth. The scale bar shows 100 or 200 µm. The organoid was rotated because medium was added between days 4 and 5.

**Figure 6:**
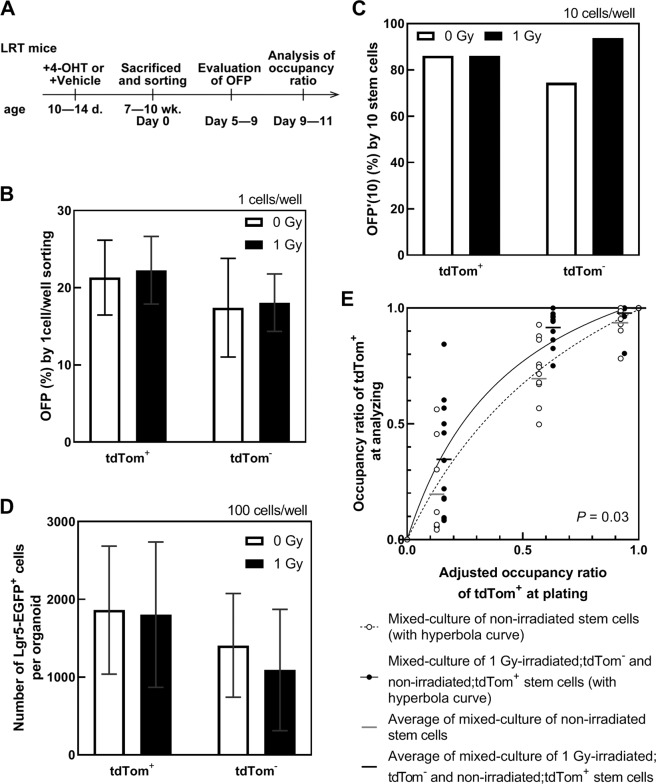
Effect of cell competition on organoid growth. (**A**) Organoid-forming procedure. (**B**) Organoid-forming potential (OFP) for one Lgr5-enhanced green fluorescent protein (EGFP)^high^ cell/well with sorting at days 5–9. Data are shown as means ± SD (n = 5). There are no statistically significant differences (*P* > 0.05, Tukey-Kramer). (**C**) OFP’(10) at days 9–10. (**D**) Numbers of Lgr5-EGFP^high^ cells in each organoid at days 9–11. Data are shown as means ± SD (n = 12–15). There are no statistically significant differences (*P* > 0.05, Tukey-Kramer). (**E**) Competition between non-irradiated stem cells themselves (white) or non-irradiated stem cells and 1 Gy-irradiated stem cells (black). The occupancy ratio at plating was adjusted by the number of Lgr5-EGFP^high^ cells in the organoid (**D**). The occupancy ratio was analyzed at days 9–11. The two hyperbola-fitted curves are significantly different (*P < 0.05).

The use of one organoid per well simplifies observations of organoid growth by eliminating the potential effects of other organoids. Under this condition, it is important to control the number of stem cells per well accurately by a direct sorting method to assess stem cell competition because one organoid can be formed from multiple stem cells in a well (Fig. 5A). The growth of organoids accelerated as an increasing number of stem cells were plated under the one organoid/well condition (Fig. 5B,C). Organoids were formed in about 20–30% wells for one stem cell/well sorting; in other words, the OFP was 20–30% (Figs. 4D and 6B). To confirm that few stem cells can grow in a mixed organoid derived from two different stem cell populations, we evaluated OFP’(10) as the number of organoids per well with 10 stem cells/well sorting (Fig. 1B). In this condition, the OFP’(10) was 80% (Fig. 6C). This method was applicable to evaluate organoid formation and cell competition between different types of cells. For example, stem and Paneth cells, duodenum and colon stem cells, irradiated and non-irradiated stem cells, and color-traced and uncolored stem cells.

We crossed Lgr5-EGFP-IRES-Cre^ERT2^ knock-in and ROSA26-tdTomato (LRT) mice, and treated them with or without 4-hydroxytamoxifen (4-OHT), to determine the cell origin and whether or not they were stem cells (Figs. 5D and 6A). This enabled us to form two-color organoids derived from different colors of stem cells isolated from different mice. Following stem cell proliferation, a disappearing population of one-color cells from the organoid was observed during the process of duodenal organoid growth (Fig. 5E). Two-color cells were adjacent and moved for growth (Movie S1). These organoids were isolated without dispase/collagenase treatment to avoid dissipation of fluorescence by cellular damage, and analyzed by fluorescence-activated cell sorting. The occupancy ratio of tdTomato^+^ (tdTom^+^) cells in the organoid was calculated with following equation:

$${\rm{occupancy}}\,{\rm{ratio}}\,{\rm{of}}\,{{\rm{tdTom}}}^{+}={{\rm{Lgr5}} \mbox{-} {\rm{EGFP}}}^{{\rm{high}}};\,{{\rm{tdTom}}}^{+}\,{\rm{stem}}\,{\rm{cells}}/{{\rm{Lgr5}} \mbox{-} {\rm{EGFP}}}^{{\rm{high}}}\,{\rm{stem}}\,{\rm{cells}}$$

Results of the analyses of two-color organoids derived from LRT mice injected with 4-OHT or sunflower seed oil (vehicle) showed that non-irradiated duodenal stem cells occupied the organoid in almost the same manner (Fig. 6E). Therefore, we assessed stem cell competition *in vitro* by forming two-color organoids derived from tdTom^+^ non-irradiated stem cells and tdTom^−^ stem cells immediately after exposure to 1 Gy of X-rays. Although the irradiated stem cells grew roughly equal to non-irradiated stem cells in monoculture (Fig. 6B–D), irradiated stem cells did not grow well in mixed culture with non-irradiated stem cells (Fig. 6E). The occupancy ratio of tdTom^+^ at plating was considered to be the expected value when the occupancies were equal. Thus, the occupancy ratio of tdTom^+^ at plating was adjusted by the difference of the mean number of Lgr5-EGFP^high^ cells in monocultured organoids (Fig. 6D) as shown in Fig. 6E.

In another experiment, tdTom^−^ 1 Gy-irradiated and tdTom^+^ 1 Gy-irradiated stem cells were mixed. The result was similar to two-color non-irradiated stem cells, but varied more widely than the non-irradiated group. In the case of a mixed culture of tdTom^−^ non-irradiated and tdTom^+^ 1 Gy-irradiated stem cells, the irradiated cells did not grow well (Fig. S1). Because stronger stem cells tended to be selected by the toxicity of 4-OHT injection (Figs. 6B,D and S2), we also evaluated stem cell competition using LRT mice treated with 4-OHT, and Lgr5-EGFP-IRES-Cre^ERT2^ knock-in × ROSA26-LSL-LacZ (LRZ) mice treated with 4-OHT (Fig. S3A). Similar to LRT mice with 4-OHT or oil injections, the growth of irradiated stem cells was suppressed by mixed culture with non-irradiated stem cells between LRT and LRZ mice with 4-OHT injection (Fig. S3B,C). In LRZ mice without any injection, irradiated stem cells grew equally, or more than, non-irradiated stem cells in monoculture (Fig. S4).

## Discussion

### Composition and substrates in the media used for stem cell cultures

Wnt3a is an essential protein for stem cell maintenance, self-renewal, and differentiation, and is produced by Paneth cells. R-spondin1 functions as a Wnt modulator. During organoid formation, R-spondin1 can substitute for Wnt3a^12^. Wnt3a and R-spondin1 conditioned media, however, did not contribute to the highest OFE in our results. Rather, CHIR-99021, a WNT activator, was necessary for highly efficient organoid formation (Fig. 2). Nevertheless, Wnt3a conditioned medium improved the OFE slightly because it contains many unknown factors beyond Wnt3a, and the OFE did not increase by adding R-spondin1 conditioned medium^13^. In another report, Lgr5-EGFP^+^ cells could not form organoids without Paneth cells even though Wnt3a conditioned medium was added to the basal medium^14^. Murine fetal enterospheres responded to Wnt stimulation at establishment, but once established could grow without R-spondin1 in the presence of the natural Wnt antagonist, DKK1, and a porcupine inhibitor that prevents Wnt secretion^15^. Thus, stem cells appear to require the Wnt activator rather than Wnt itself, and R-spondin1 is effective at only the early phase.

Our results demonstrate that Thiazovivin, Y-27632, and CHIR-99021 are more important for organoid formation than Wnt3a and R-spondin1 at the time of stem cell isolation and culture (Fig. 2). This is consistent with the report that medium containing Thiazovivin and CHIR99021 increased the OFE more than medium with Wnt3a and R-spondin1^13^. Moreover, Thiazovivin and Y-27632 are Rho-associated coiled-coil forming kinase (ROCK) inhibitors that prevent anoikis (i.e., apoptosis induced by the lack of cell and matrix attachments). Thus, single stem cells are sensitive to death induced by damage at the time of isolation from crypts.

When intestinal stem cells are cultured in flat-bottomed cell culture plates, organoids cannot form in medium with 1% Matrigel. Thus, cells are generally embedded into 10% of Matrigel. Furthermore, treatment with dispase/collagenase is necessary to extract the organoids from this medium. In this study, we found that organoids can be cultured in medium with 1% Matrigel when using plates with V-shaped wells (Fig. 4). Importantly, the organoids can be obtained without dispase/collagenase treatment. Therefore, our 1% Matrigel culture method has advantages including fixation immediately after treatment with chemicals and radiation, addition of some reagents, and viral transfections.

### OFP

Improvement of the efficiency of stem cell cultures and organoid formation enabled us to assess the OFP by one cell/well direct sorting. This procedure was not reported previously, although one cell-derived organoid formation by a dilution method was reported in 2009^10^. When a large number of cells were sorted into a well, the OFE of Lgr5-EGFP^high^ cells isolated enzymatically from organoids was higher than that of cells isolated from tissues. Therefore, the OFP of Lgr5-EGFP^high^ cells can be improved if cell damage caused by tissue isolation or culture stress is further reduced. Other advantages of our new method of one stem cell/well sorting include:

- The OFP can be evaluated for a stem cell using information from fluorescence-activated cell sorting about forward scatter (information of cell size), side scatter (information of the cell’s internal structure), fixable viability dye, and EGFP.
- Single organoids can be followed and observed easily for a long time.
- Organoids are available for mutation analyses by whole genome or exome sequencing because a single stem cell grows into an organoid via clonal proliferation.
- Many organoids can be formed by using a small number of mice; this can improve animal welfare.

Recently, functional human gut organoids, including neural cells with peristalsis, were reported^16,17^. However, mouse organoids derived from adult stem cells remain essential for comparing data sets obtained from *in vivo* and *in vitro* experiments. For example, in discussing the health effects of ionizing radiation based on *in vitro* experimental results, extensive data from *in vivo* animal experiments stored for many years can be used, whereas available data from healthy humans are limited.

### Cell competition in organoids as an intestinal stem cell pool

Normal intestinal stem cells compete stochastically by self-renewal *in vivo*^4,18–22^. Our *in vitro* results (Figs. 6E and S1C) are consistent with these *in vivo* data. In addition, one characteristic of our method is an Lgr5 tracing system using the stem cell marker, Lgr5-cre, rather than Ah*cre*^ERt^, which is used in many studies as a marker of intestinal epithelium cells, including both stem and differentiated cells.

In the case of non-neutral competition between normal and transformed cells having *Kras* and *APC* mutations, they have differing levels of fitness to occupy a niche in the crypt^22^. Cells with mutations in *ß-catenin* are easier to eliminate by normal cells in normal intestinal crypts compared to *APC* mutated cells^23^. In the intestine of *Rosa26-LacZ/Ah-Cre/Ascl2*^*floxed/floxed*^ mice, all cells are visually recombined except for long-lived Paneth cells at five days post injection. However, all crypt cells are replaced by wild-type cells at 20 days post injection^24^. The rapid reappearance of wild-type epithelium suggests a strong selective pressure favoring the few remaining Ascl2^+^ epithelial Lgr5 stem cells. In addition, wild-type crypts that escape conditional deletion of *Sox9* and contain abundant Paneth cells rapidly replace mutant crypts by crypt fission^25^. Cell competition is observed not only in *in vivo* studies, but also in *in vitro* and *ex vivo* studies. Transformed cells are eliminated from intestinal organoids when they are surrounded by normal cells *ex vivo*^7,8^. Overall, we considered that cell competition is constantly occurring to maintain homeostasis in the intestine.

High doses of ionizing radiation are well-known as mutagenic. However, competition between irradiated and non-irradiated stem cells has not been well-investigated. Activation of tumor suppressor p53 signaling occurs in mouse intestine after irradiation^26^. In the current study, we established a high efficiency two-color organoid culture system by mixing stem cells expressing different fluorescent colors (Figs. 5 and 6). Using this system, we assessed stem cell competition between X-ray-irradiated and non-irradiated intestinal stem cells plated just after irradiation. The results showed that growth of irradiated stem cells was partially suppressed in the organoid in the presence of non-irradiated stem cells. This suggests that X-ray-irradiated stem cells were outcompeted by non-irradiated cells, at least partly. Moreover, our results are important to consider when evaluating the health risk of low-dose and low-dose rate radiation exposures where irradiated and non-irradiated stem cells co-exist in the stem cell pool. Stem cell competition may act as a tissue-based quality control mechanism to prevent radiation-induced carcinogenesis under low-dose and low-dose rate irradiation conditions.

## Methods

### Mice

Lgr5-EGFP-IRES-Cre^ERT2^ (B6.129P2-Lgr5^tm1(cre/ERT2)Cle^/J; JAX mice #008875), ROSA26-LSL-LacZ (B6.129S4-Gt(ROSA)26Sor^tm1Sor^/J; JAX mice #003474), and ROSA26-tdTomato (B6.Cg-Gt(ROSA)26Sor^tml4(CAG-tdTomato)Hze^/J; JAX mice #007914) mice were purchased from the Jackson Laboratory. Mice were bred in a conventional clean facility of the Central Research Institute of Electric Power Industry (CRIEPI) at a controlled temperature (24 ± 2 °C) and humidity (45 ± 5%), with a 12-h light–dark cycle, and ad libitum access to γ-sterilized food (CLEA Japan) and filter-sterilized deionized water. In this paper, Lgr5-EGFP-IRES-Cre^ERT2^ × ROSA26-LSL-LacZ mice are called LRZ mice and Lgr5-EGFP-IRES-Cre^ERT2^ × ROSA26-tdTomato mice are called LRT mice. All animal experiments were approved by the Animal Research and Ethics Committee at CRIEPI and were performed in accordance with the guidelines for animal care in Japan.

### Tissue preparation and cell sorting

Details for tissue preparation were described previously^27^. In brief, to obtain tdTomato^+^ crypts, 4-OHT (Sigma-Aldrich) was dissolved in sunflower oil at 10 mg/mL and injected intraperitoneally into LRT mice (3 mg/40 g body weight). To obtain tdTomato^−^ crypts, sunflower seed oil without 4-OHT was injected intraperitoneally into LRT mice or 4OHT was injected intraperitoneally into LRZ mice at 10–14 days or 6–9 weeks of age. LRT or LRZ mice were sacrificed at 7–10 weeks of age (i.e., 5–8 weeks or 8 days after injection of 4-OHT or sunflower seed oil), and the duodenum (10–12 cm length of proximal intestine) was harvested and rinsed three times with ice-cold phosphate-buffered saline without Ca^2+^ and Mg^2+^ (PBS (−)). Then, the intestinal lumens were opened longitudinally and the villi scraped using a coverslip, followed by washing three times in ice-cold PBS (−). Tissue fragments were cut into 2–3 mm pieces and suspended in PBS (−) containing 2% fetal bovine serum, washed once with PBS (−), and incubated in 50 mM EDTA/PBS (−) for 30 min at 4 °C (or on ice) on a rocking platform to dissociate crypts from the intestinal tissue. Crypts were passed through a 70-μm cell strainer, washed once with ice-cold PBS (−), and treated with TrypLE Express (Life Technologies) containing YTC (10 μM Y-27632 (Sigma-Aldrich), 2 μM Thiazovivin, and 2.5 μM CHIR99021 (Stemgent)) for 30 min at 37 °C to dissociate cell-to-cell attachments. To prepare a single-cell suspension, dissociated cells were passed through 40- and 20-μm strainers, then resuspended in PBS (−) containing 2% fetal bovine serum and YTC. Cells were isolated using a MoFlo Astrios EQ cell sorter (Beckman Coulter). Flow cytometry data were analyzed using Summit Software (Beckman Coulter). To exclude dead cells, samples were stained with Fixable Viability Dye eFlour450 (#65-0863-14, ThermoFisher Scientific) before cell sorting.

### *In vitro* culture of single crypt cells

Isolated single stem cells were cultured in organoid medium (Advanced Dulbecco’s modified Eagle’s/F12 medium with 100 × GlutaMax and 1 M HEPES (ThermoFisher Scientific) or IntestiCult Organoid Growth Medium (mouse) (Stemcell Technologies) containing 1 × penicillin/streptomycin, 1 × N2, 1 × B27, 50 ng/mL murine epidermal growth factor (Life Technologies), 1 mM N-acetylcysteine (Sigma-Aldrich), 100 ng/mL murine Noggin (Peprotech)). IntestiCult Organoid Growth Medium (mouse) described above was used for all experiments except for Fig. 2 where it also contained R-spondin1 and Wnt3a conditioned media. Both YTC, and or 10% Matrigel, were added to the medium immediately before dispensing it into each well of the cell culture plate. For OFE evaluation, stem cells were sorted into 10% Matrigel-containing medium using plates with flat-shaped wells. For “one cell/well direct sorting” or forming mixed organoids, stem cells were sorted into 1% Matrigel-containing medium using plates with V-shaped wells. Thereafter, freshly prepared organoid medium was added every 3–4 days. Cells were cultured in a humidified CO_2_ incubator at 37 °C. The number of organoids was counted 5–9 days after plating by visual observation under a phase-contrast microscope with a 4 × objective lens(Olympus IX71/ DP71).Organoid images were taken using an Olympus IX71/ DP71 and Olympus IX71/ Sony α NEX-5R. Sectioning and time-lapse imaging were performed with a BZ-X700 fluorescence microscope (Keyence). Images of single organoids per well were captured by an Olympus IX71/ DP71 microscope. The captured images were analyzed by Image J software (National Institutes of Health).

### Irradiation

Immediately after sorting, Lgr5-EGFP^high^ cells were irradiated by an MBR-1520R4 X-ray generator (Hitachi Power Solutions) operated at 150 kV with 20 mA and a 0.5 mm Al plus 0.3 mm Cu filter at a dose rate of 0.6 Gy/min.

### Isolation of cells from organoids for analyzing organoid passage

Organoids were dissociated with TrypLE Express + YTC for 10 min at 37 °C to prepare single cell suspensions.

### Statistical analyses

Data are presented as means ± standard deviation of the mean. Differences between two groups were determined by Student’s t test using Microsoft Excel and GraphPad Prism 8.2, The Tukey-Kramer test was used for comparison of multiple groups with GraphPad Prism 8.2. A *P* value less than 0.05 was considered significant.

## Supplementary information

Movie S1 mixed organoid from Day 2

Supplementary information

## References

[1] Cheng H, Leblond CP (1974). Origin, differentiation and renewal of the four main epithelial cell types in the mouse small intestine. V. Unitarian Theory of the origin of the four epithelial cell types. Am. J. Anat..

[2] Barker N, Clevers H (2007). Tracking down the stem cells of the intestine: strategies to identify adult stem cells. Gastroenterology.

[3] Barker N (2007). Identification of stem cells in small intestine and colon by marker gene Lgr5. Nature.

[4] Snippert HJ (2010). Intestinal crypt homeostasis results from neutral competition between symmetrically dividing Lgr5 stem cells. Cell.

[5] Barker N (2009). Crypt stem cells as the cells-of-origin of intestinal cancer. Nature.

[6] Vivarelli S, Wagstaff L, Piddini E (2012). Cell wars: regulation of cell survival and proliferation by cell competition. Essays Biochem..

[7] Yamauchi H (2015). The cell competition-based high-throughput screening identifies small compounds that promote the elimination of RasV12-transformed cells from epithelia. Sci. Rep..

[8] Kon S (2017). Cell competition with normal epithelial cells promotes apical extrusion of transformed cells through metabolic changes. Nat. Cell Biol..

[9] Stine RR, Matunis EL (2013). Stem cell competition: finding balance in the niche. Trends Cell Biol..

[10] Sato T (2009). Single Lgr5 stem cells build crypt-villus structures *in vitro* without a mesenchymal niche. Nature.

[11] Yamauchi M (2014). A novel *in vitro* survival assay of small intestinal stem cells after exposure to ionizing radiation. J. Radiat. Res..

[12] Yui S (2012). Functional engraftment of colon epithelium expanded *in vitro* from a single adult Lgr5(+) stem cell. Nat Med.

[13] Wang F (2013). Isolation and characterization of intestinal stem cells based on surface marker combinations and colony-formation assay. Gastroenterology.

[14] Yilmaz OH (2012). mTORC1 in the Paneth cell niche couples intestinal stem-cell function to calorie intake. Nature.

[15] Fordham RP (2013). Transplantation of expanded fetal intestinal progenitors contributes to colon regeneration after injury. Cell stem cell.

[16] Uchida H (2017). A xenogeneic-free system generating functional human gut organoids from pluripotent stem cells. JCI Insight.

[17] Workman MJ (2017). Engineered human pluripotent-stem-cell-derived intestinal tissues with a functional enteric nervous system. Nat. Med..

[18] Klein AM, Simons BD (2011). Universal patterns of stem cell fate in cycling adult tissues. Development.

[19] Lopez-Garcia C, Klein AM, Simons BD, Winton DJ (2010). Intestinal stem cell replacement follows a pattern of neutral drift. Science.

[20] Simons BD, Clevers H (2011). Stem cell self-renewal in intestinal crypt. Exp. Cell Res..

[21] Simons BD, Clevers H (2011). Strategies for homeostatic stem cell self-renewal in adult tissues. Cell.

[22] Vermeulen L (2013). Defining stem cell dynamics in models of intestinal tumor initiation. Science.

[23] Song JH (2014). The APC network regulates the removal of mutated cells from colonic crypts. Cell reports.

[24] van der Flier LG (2009). Transcription factor achaete scute-like 2 controls intestinal stem cell fate. Cell.

[25] Sato T (2011). Paneth cells constitute the niche for Lgr5 stem cells in intestinal crypts. Nature.

[26] Otsuka K, Suzuki K (2016). Differences in Radiation Dose Response between Small and Large Intestinal Crypts. Radiat. Res..

[27] Otsuka K (2013). Ionizing radiation leads to the replacement and de novo production of colonic Lgr5(+) stem cells. Radiat. Res..

