# Supplementary material for: An Efficient Intestinal Organoid System of Direct Sorting to Evaluate Stem Cell Competition in Vitro
Source: Sci Rep. 2019 Dec 30;9:20297. doi: 10.1038/s41598-019-55824-1 (PMC6937314; doi:10.1038/s41598-019-55824-1)
Supplement: Supplementary file 2 — Supplementary information [file 41598_2019_55824_MOESM2_ESM.docx]

**Supplementary Information**

**An Efficient Intestinal Organoid System of Direct Sorting to Evaluate Stem Cell Competition in Vitro**

Yuki Fujimichi,^1,*^ Kensuke Otsuka, ^1^ Masanori Tomita, ^1^ and Toshiyasu Iwasaki^1^

^1^Radiation Safety Research Center, Nuclear Technology Research Laboratory, Central Research Institute of Electric Power Industry (CRIEPI), 2–11–1 Iwado Kita, Komae, Tokyo 201–8511, Japan

*Correspondence: f-yuki@criepi.denken.or.jp

**Table S1. Concentrations of Wnt3a and RSPO1 in the media**


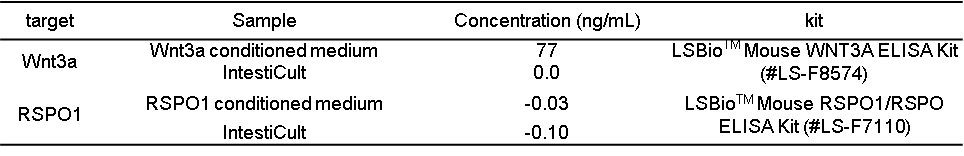





**Figure S1. Cell competition in growing organoids**

(A) Organoid-forming procedure. (B) Organoid forming potential (OFP) at days 5-7 for 1 stem cell/well sorting. Data are shown as means ± SD (n = 3). There is no significant difference (*P* > 0.05, Tukey-Kramer). (C) Stem cell competition between non-irradiated stem cells (white) or 1 Gy-irradiated stem cells (gray) at days 10-12. There is no significant difference between two hyperbola-fitted curves (*P* = 0.18). (D) Stem cell competition between non-irradiated stem cells (white) or 1 Gy-irradiated;tdTom^+^ and 1 Gy-irradiated;tdTom^-^ stem cells (black) at days 10-12. The occupancy ratio at plating was adjusted by the OFP (panel B). There is no significant difference between two hyperbola-fitted curves (*P* = 0.47). 4-OHT, 4-hydroxytamoxifen





**Figure S2. 4-Hydroxytamoxifen (4OHT) injection reduces stem cells in crypt cells.**

LRT mice injected with 4-OHT or vehicle eight days before sacrifice. Percentages of stem cells were compared after population was selected the live crypt base cells, and discriminated for doublets. Data are expressed as means ± SD (n = 3). There is no significant difference (*P* = 0.12, Student’s t-test).





**Figure S3. Cell competition in growing organoids from mice injected with 4-hydroxytamoxifen (4OHT).**

(A) Organoid-forming procedure. (B) Organoid forming potential (OFP) at days 6-8 for 1 stem cell/well sorting in LRT and LRZ mice. Data are expressed as means ± SD (n = 5) (**P* < 0.05, Student’s t-test). (C) Stem cell competition between non-irradiated stem cells (white) or non-irradiated stem cells and 1 Gy-irradiated stem cells (black). The occupancy ratio was evaluated at day 13. The occupancy ratio at plating was adjusted by the OFP (panel B). There is no significant difference between two hyperbola-fitted curves (*P* = 0.15).





**Figure S4. Cell competition in growing organoids as a model of the intestinal stem cell pool.**

(A) Numbers of enhanced green fluorescent protein (EGFP)^high^ cells in the organoids. (B) Numbers of cells in the organoids. (C) Organoid size (mm^2^). (A-C) LRZ mice without any injection. There is no significant difference between the control and 1 Gy-irradiated groups at any day (*P* > 0.05, Student’s t-test).


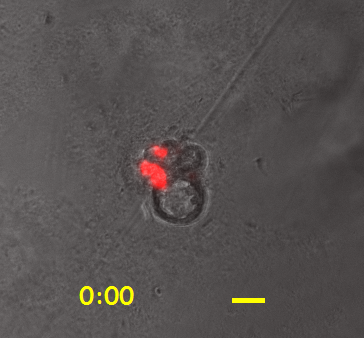


**Movie S1**

Mixed-organoid from Day 2.
